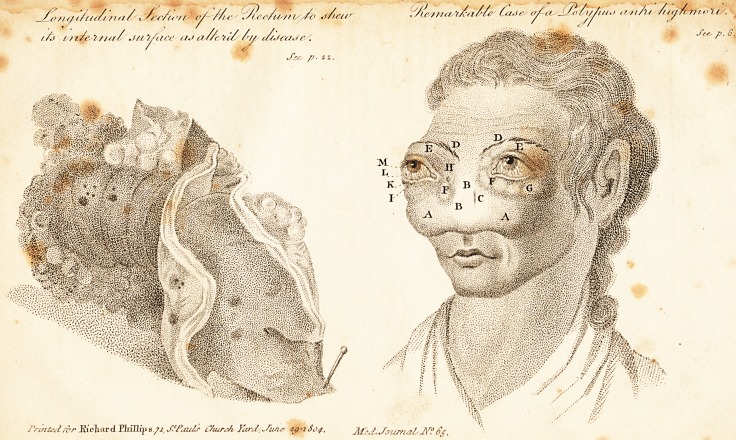# Remarkable Case of a Polypus Antri Highmori
*This case is described in Dr. Eichhorn's Inaugural Dissertatio de Polypis, &c. Gottingen, 1804.


**Published:** 1804-07-01

**Authors:** 


### REMARKABLE CASE OF A POLYPUS ANTRI HIGHMORI.*

[ With an Engraving. ]

A Peasant's wife, who lived in a village near Gottingen, about fifty years of age, born of healthy parents, was accidentally struck, when ten years old, in the right cheek with the tooth of a rake, which penetrated nearly one inch deep. She lost a great deal of blood, but the wound closed a short time after. From this period however she was often attacked with acute pains in the maxilla superior, particularly during her menstruation, which occurred for the first time, sparingly, in her nineteenth year. The right cheek began gradually to swell; the pains continued from time to time, and alternated with other rheumatic complaints. When the pains were violent and the cheek much swoln and red, she used to apply cataplasms of aromatic herbs, which greatly relieved her. In her twenty-seventh year she married a peasant, by whom she had two children, who, to the present day, enjoy very good health and are brisk young men. All the time she suffered more or less violent pains, particularly in winter, and the swelling of the cheek proceeded very slowly, till after her last child-birth, when she perceived that the tumour of the cheek increased more evidently; the eyes became a little prominent, and the pains more frequently returned. In this state she remained for many years; but after the cessation of fluxus mensium, which happened in her forty-sixth year, the condition of the patient became considerably worse. The bulb of the right eye protruded from the orbit, without any diminution of sight, the teeth of the upper and lower jaw fell out, the respiration by the nose was much impeded, and the power of smelling quite gone. Speech and manducation became more difficult, and the tumour, protrusion of the eye, &c. extended to the left side. All this time she had never taken the advice of a medical man, but had committed herself to the care of quacks and old women.

In June, 1803, the local affections being exceedingly increased, the pains almost insupportable, and the patient attacked by febrile symptoms, which confined her to her bed, the husband walked to Gottingen, in order to consult Dr.

* This case is described in Dr. Eichhorn's Inaugural Dissertatio de Polypis, &c. Gottingen, 1804.

*Longitudinal Section of the Rectum to shew its internal surface as alter'd by disease.*

*See p. 22.*

*Remarkable Case of a Polypus antri highmori.*

*See p. 6.*

*Printed for* Richard Phillips, *71, St. Paul's Church Yard, June 29. 1804.*

Dr. Noehden, who ordered several remedies, which he thought proper, according to the imperfect relation of this man, who only told him her present condition, without mentioning a syllable of the progress and beginning of her chief complaint. The remedies however operated so well, that within a few weeks the most urgent symptoms were removed, the fever subdued, and the patient able to quit the bed. The diseases of which she now chiefly complained were a dimness of sight, and pains in the upper jaw, but they were much less violent than before. As these symptoms continued, notwithstanding the most efficacious remedies, Dr. N. desired the patient to come to town, that he might see her, which he had not hitherto had an opportunity of doing. The Doctor was surprized at the great deformity of the face; and on examining the patient, and exploring the state in which she was, soon found that she ought to have recourse to the assistance of surgery, and therefore recommended her to Dr. Langenbeck, an able surgeon of that place, at whose house Dr. Eichhorn saw and examined the patient.

The chief symptoms observable at that time were, 1. A swelling of both sides of the maxilla superior, which was hard and a little red. 2. The nasal bones flat. 3. In both cavities of the nose a polypus of a red colour. 4. The bulbs of the eyes much protruded, and the sight diminished. 5. The cornea pellucid. 6. Saccus lacrymalis tumid and inflamed. 7. Puncta lacrymalia shut. 8. A small tumour in the region of the glandula lacrymalis. 9. A continual watering of the eyes. 10. The palate was depressed, rather convex, and yielded like a tumour to the pressure of the finger. Except these symptoms, the patient found herself pretty well; appetite good, pulse soft, and not very frequent. The polypi in the nose induced the present gentlemen to ascribe the above-mentioned deformity, and the attending symptoms, to a similar excrescence which had taken place in the antrum Highmori. On having sufficiently considered the case, Dr. Langenbeck proposed an operation, as the only expedient to save the patient's life, however doubtful its success might seem to be. He intended to have made an incision in the palate, and by this way endeavour to extract the contents of the antrum Highmori; but the patient could not by any means be persuaded to submit to this operation, and returned home without farther medical assistance. Under the care of quacks and old women, to whom the patient had again recourse, the disease evidently in-

 creased creased in the course of a few months, so that, when Dr. Eichhorn visited her in December, 1803, in order to observe the progress of the disease, and to obtain a drawing of her face, the disease had so far proceeded, as is represented in the annexed engraving.

EXPLANATION OF THE PLATE.

AA. The tumour of the maxilla superior very considerable.

BB. The nose formed with the maxilla one even tumour, and could not be distinguished. The bones of these parts soft and yielding to pressure.

C. A cicatrice where a barber had made an incision.

DD. The eye-brows, much drawn from one another.

EE. The bulbs of the eyes enormously protruded and total blindness of both eyes.

FF. The right saccus lacrymalis very tumid.

G. The glandula lacrymalis extremely swoln, and extending in form of a bag from the upper eye-lid to the temples.

H. An opening of the right saccus lacrymalis, from which a thin matter copiously issued.

I. Puncta lacrymalia extended, and discharging a great deal of matter.

K. The glandulæ meibomianæ discharged a similar matter.

L. The blood-vessels of the membrana conjunctiva much extended, and the conjunctiva tumid, red, and hanging over the inferior eye-lid.

M. The iris, protruded by the pressure of the crystalline lens, and much distended, seemed to adhere to the interior coating of the cornea. The pupil immoveable.

These symptoms were attended with an insupportable stench, which surrounded the patient, a great emaciation of the body, extreme weakness, want of appetite, sleepless nights, pains, and excruciating heat. Although Dr. Eichhorn offered his assistance, in order to mitigate at least the most excruciating pains, she entirely refused to take any medicine. In this miserable condition she continued for some weeks; and when he saw her last, part of the polypus antri Highmori had come out of the opening H. hanging down to A. A few days after she died.

## Figures and Tables

**Figure f1:**